# Amelogenin-Derived Peptides in Bone Regeneration: A Systematic Review

**DOI:** 10.3390/ijms22179224

**Published:** 2021-08-26

**Authors:** Antonino Fiorino, Alessandro Marturano, Giacomo Placella, Edoardo Staderini, Lorena Igual Domingo, Giuliano G. Cerulli, Roberto Tiribuzi, Paolo Blasi

**Affiliations:** 1 Department of Dentistry, Catholic University of Sacred Heart, 00168 Rome, Italy; fiorinodr.antonino@gmail.com (A.F.); edoardo.staderini@unicatt.it (E.S.); 2 GBR Academy, 50122 Florence, Italy; alessandro.marturano@hotmail.it; 3 Department of Traumatology and Orthopedics, San Raffaele Hospital, 20132 Milan, Italy; giacomo.placella@gmail.com; 4 Department of Neurosciences, Dentistry Section, University of Padova, 35121 Padova, Italy; lorenaigualdomingo@gmail.com; 5 Biology and Regenerative Medicine Laboratory, Translational Research Institute for the Locomotor System Nicola Cerulli-LPMRI, 52100 Arezzo, Italy; giulianocerulli@gmail.com; 6 Department of Pharmacy and Biotechnology, University of Bologna, 40127 Bologna, Italy; p.blasi@unibo.it

**Keywords:** LRAP, TRAP, synthetic peptide, SP, amelogenin C11 peptide, regenerative medicine, bone diseases, biomineralization

## Abstract

Amelogenins are enamel matrix proteins currently used to treat bone defects in periodontal surgery. Recent studies have highlighted the relevance of amelogenin-derived peptides, named LRAP, TRAP, SP, and C11, in bone tissue engineering. Interestingly, these peptides seem to maintain or even improve the biological activity of the full-length protein, which has received attention in the field of bone regeneration. In this article, the authors combined a systematic and a narrative review. The former is focused on the existing scientific evidence on LRAP, TRAP, SP, and C11’s ability to induce the production of mineralized extracellular matrix, while the latter is concentrated on the structure and function of amelogenin and amelogenin-derived peptides. Overall, the collected data suggest that LRAP and SP are able to induce stromal stem cell differentiation towards osteoblastic phenotypes; specifically, SP seems to be more reliable in bone regenerative approaches due to its osteoinduction and the absence of immunogenicity. However, even if some evidence is convincing, the limited number of studies and the scarcity of in vivo studies force us to wait for further investigations before drawing a solid final statement on the real potential of amelogenin-derived peptides in bone tissue engineering.

## 1. Introduction

A wide range of pathological phenomena can be directly or indirectly responsible for skeletal tissue loss. Many of the generated defects, known as critical size defects (CSDs), do not heal spontaneously. The best option to treat CSDs is an autologous bone graft that possesses the three main features needed in bone regenerative medicine: osteogenic, osteoinductive, and osteoconductive properties. Unfortunately, autologous bone graft is rarely used due to a number of drawbacks, such as the requirement of a second surgical procedure with serious risks of infection at the donor site and the generation of significant pain.

Regenerative medicine and tissue engineering offer alternative strategies for the treatment of CSDs. In most of the approaches proposed, molecules capable of stimulating cell migration, recruitment, proliferation, and differentiation, as well as biomineralization, play a pivotal role in the formation of de novo bone tissue. Platelet-derived growth factors, insulin-like growth factors, transforming growth factors, and bone morphogenic proteins are examples of biomolecules investigated so far [1]. However, several efforts are still ongoing to individuate effective and safe bone morphogenic biomolecules. Among the biological macromolecules under investigation, amelogenins (AMG) represent an extremely interesting family of proteins with the above-mentioned characteristics for which the bone morphogenic properties are still matter of debate.

AMG are structural proteins secreted by the inner enamel epithelium during tooth development and represent about 90% of the enamel matrix proteins. Together with proline-rich proteins, such as ameloblastins, enamelins, and tuftelins, they direct the mineralization of enamel to form the highly organized rod matrix and the interrod crystals. AMG are highly conserved proteins, which are known to be essential in the formation of enamel [2].

AMG-based preparations were first proposed in the dermatological field for the treatment of burns and, only later, in dentistry. The first AMG formulation marketed for periodontal tissue regeneration procedures was Emdogain^®^. The product contains a mixture of animal enamel matrix derivatives embedded in an alginate propylene glycol hydrogel. After 20 years, the use of Emdogain^®^ in periodontal regeneration procedures has shown a statistically significant improvement in the recovery of the periodontal ligament, cement, and alveolar bone [3].

Although for over four decades, AMG was considered a specific enamel protein expressed in periodontal tissues, such as cementoblasts, periodontal ligament (PDL) cells, or Hertwig’s epithelial root sheath (HERS) [4,5,6,7], its expression has also been reported, at a lower level, in non-dental cell types, such as stem cells, bone cells, brain, and other soft tissue [8,9,10]. Of interest are some observations suggesting that specific AMG splicing products may function as epithelial-mesenchymal or mesenchymal-mesenchymal signaling molecules [11,12,13,14]. In the late 1960s and early 1970s, two articles showed the osteoinductive potential of decalcified enamel and dentin extracts in ectopic sites [15,16]. This phenomenon was attributed to the presence of peptides with chondro-/osteoinduction properties derived from AMG gene splicing [17]. AMG-derived peptides are formed by alternative splicing or proteolytic cleavage of the ~20 kDa full-length protein [18,19].

In this review, we discuss the structural and functional properties and the possible use in bone regeneration of natural and synthetic AMG-derived peptides. The article includes: a systematic review on the use of AMG-derived peptides in bone regeneration and a narrative review on the structure and function of AMG and AMG-derived peptides. The systematic review, which is focused on leucine-rich AMG peptide (LRAP), tyrosine-rich AMG peptide (TRAP), synthetic peptide (SP), and AMGC peptide (AMG-CP or C11), attempts to answer the following questions: Do AMG peptides have osteoinductive capabilities? Which performs the best? Could their use in bone regenerative medicine/tissue engineering procedures be advantageous? The narrative part attempts to answer the following questions: What are AMG and what are their structures? What are the coding genes? Which cells synthesize AMG? What function do they have? Are they really useful in the regenerative field and on which tissues?

## 2. Methods

### 2.1. Search Strategy and Literature Screening

For the systematic review, two online databases (MEDLINE and Cochrane Library) were consulted for the publication period from 1980 to 20th January 2020 and the following search terms were used: “Amelogenin Peptide” OR “Leucine-rich amelogenin peptide” OR “Tyrosin rich amelogenin peptide” OR “LRAP” OR “TRAP” OR “Amelogenin Synthetic Peptide” OR “Amelogenin C Peptide” OR “Amelogenin Splice Product” OR “Amelogenins” AND “Osteoblast” OR “Bone” OR “Mineralized Tissue” OR “Mineral Nodule” OR “Tissue Regeneration” OR “Regenerative Medicine” OR “Tissue Engineering” (mp = title, original title, abstract, name of substance, mesh subject heading). This systematic review was performed with PRISMA (Preferred Reporting Items For Systematic Reviews and Meta-Analyses) statement.

For the narrative review, online databases and the publication period remained unchanged, while the search term combinations were: “Amelogenins” OR “Amelogenin Peptide” OR “Enamel Matrix Proteins” OR “Amelogenin Splice Product” OR “Leucine-rich amelogenin peptide” OR “Tyrosin rich amelogenin peptide” OR “LRAP” OR “TRAP” OR “Amelogenin C Peptide” (mp = title, original title, abstract, name of substance, mesh subject heading).

### 2.2. Exclusion Criteria

Only studies involving bone formation evaluations in vitro and/or in vivo using amelogenin peptides were considered. The following exclusion criteria were applied:

- Articles not written in English;
- Letters;
- Duplicate publications (the article with the most recent data was preferred);
- Dental development;
- Guided tissue regeneration approach;
- Lacking mineral deposition or histomorphometric analysis;
- Bone formation not investigated.

### 2.3. Study Selection and Data Extraction

The selection procedures were conducted independently by three authors (AF, AM, and RT), and disagreements were resolved by full text analysis and a discussion session. The selection by titles and abstracts included articles that reported unclear or incomplete data in the full text analysis to minimize the possibility of excluding relevant articles. Selected publications, including already published reviews [11,20,21,22,23], were screened, and the bibliographies of all selected articles were checked so that other potentially relevant studies were also included in the analysis.

A specific dataset was created which included the following information: author, year of publication, type of study, objectives, type of cells used, type of peptide and concentration, culture medium, timing, presence of a control group and characteristics, outcomes, methods of analysis, and results. In case of incomplete or unclear information, the article’s corresponding author was contacted. The articles were classified and described on the basis of the AMG peptides used.

## 3. Results and Discussion

### 3.1. Systematic Review

**Figure 1:**
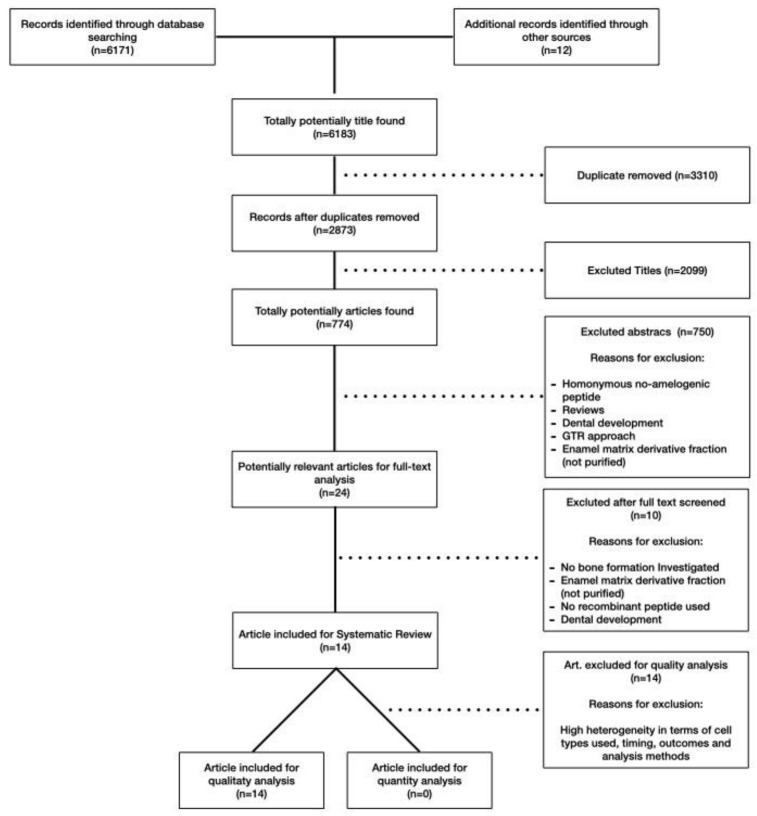
PRISMA flow chart and search strategy.

Electronic research identified 6171 studies, while a further 12 were collected by a manual search and references from selected articles and reviews. The selection sequence consisted of the evaluation of the title (2873 articles), of the abstract (774 articles), and subsequently of the complete text (24 articles) (Figure 1). Of the latter, 10 [24,25,26,27,28,29,30,31,32,33] were excluded due to the descriptive analysis (Table 1), while 14 studies met the inclusion criteria (Table 2). In the 14 studies selected [8,34,35,36,37,38,39,40,41,42,43,44,45,46], it was not possible to perform a quantitative analysis due to major differences in terms of the cell model, peptide concentrations, time points, and methodologies employed (Table 2). Among the included papers, there were no clinical trials, 13 were in vitro [8,34,35,36,37,38,39,41,42,43,44,45,46], and 1 was in vivo [40]. Only one article tested TRAP [38], five LRAP [8,34,35,36,37], six SP [39,40,41,42,43,44], and two the C11 peptide [45,46].

Fifty percent of the selected articles evaluated, simultaneously, the effect of the various peptides on osteoblastic differentiation, deposition of mineral crystals, and cell proliferation [35,37,39,41,42,43,46].

### 3.2. Amelogenins

#### 3.2.1. Proteins and Genes

AMGs were described for the first time by Eastoe in 1965 [47], but the first amino acid sequence (bovine) was reported by Takagi et al. only in 1984 [48]. AMG is encoded in the X and Y chromosomes, both genes are expressed, and the transcript undergoes alternative splicing leading to different AMG isoforms. In humans, the AMG protein is mainly encoded (approximately 90%) by the X chromosome. Lau and coworkers identified the 7 exons AMG genes on sex chromosomes in humans, on the X and Y chromosomes by Southern blot analysis of human–rodent cell hybrid [49]. In the following years, amino acid sequences from several mammalian species were identified [50,51,52]. Nakahori et al. [53] reported two genomic sequences of AMG genes; one copy of the AMG genes was located on the distal short arm of the X chromosome in the Xp22.1-Xp22.3 region (*AMELX*), while the second copy was near the centromere of the Y chromosome in the Yp11.2 region (*AMELY*). The *AMELX* is localized within the intron 1 of the *ARHGAP6* gene, with an opposite orientation to the nested gene [54]. In humans, the homology between *AMELX* and *AMELY* genes is about 88.9%, while between the two cDNA sequences, it is about 91%. 

Globally, 90% of all transcripts derive from the 7 exons X-linked gene. Translation starts from exon 2, with exon 4 being frequently skipped. More than 15 different mRNAs were described from *AMELX* gene.

The mature human AMG from the X chromosome, skipping exon 4, is a protein of 19.8 kDa with 180 amino acid residues [55,56], while the Y chromosome produces a 21.6 kDa protein with 190 amino acids (data from www.uniprot.org, accessed on 30 July 2021).

Differences between *AMELX* and *AMELY* are used in sex determination. *AMELX* intron 1 contains a 6 bp deletion relative to intron 1 of the *AMELY*. This can be detected by polymerase chain reaction (PCR) of intron 1 [57,58]. Starting from the consideration that human teeth have been considered as a prime choice in determining the identity of an individual, recently, several works have described the use of AMG genes in forensics. Dutta et al. showed that pulpal tissue along with degenerating odontoblastic processes yield a sufficient amount of DNA for gender determination with maximum accuracy by PCR [59]. They have also shown the reliability of the test even in teeth exposed to extreme environmental conditions or insults [60].

**Figure 2:**
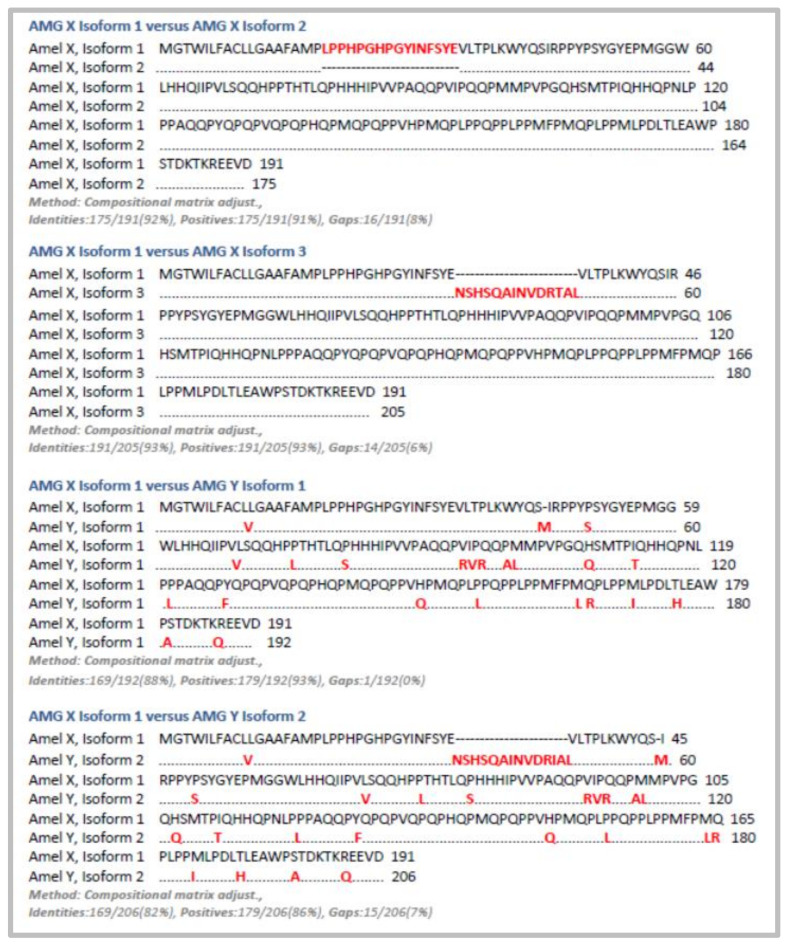
BLAST pairwise with dots for identities between Amelogenin x isoform 1 and the other four isoforms.

So far, five different AMG isoforms and numerous peptides have been described. Figure 2 reports the BLAST comparative analysis between Amelogenin X isoform 1 (Swiss prot: Q99217) and the other four isoforms (Amelogenin X isoform 2, Swiss prot: Q99217-2; Amelogenin X isoform 3, Swiss prot:Q99217-3; Amelogenin Y isoform 1, Swiss prot: Q99218-1; Amelogenin Y isoform 2, Swiss prot: Q99218).

AMG are part of a heterogeneous family of proteins that undergo proteolytic degradation, resulting in enamel formation. According to the amino acid physico-chemical characteristics, the primary structure can be organized into three main domains, including the signal peptide at the N-terminal. The first domain, TRAP, is a highly conserved hydrophobic domain (aa 1–45) that includes the tyrosyl binding motif (aa 34–45) and the kallikrein 4 (KLK4) cleavage site between amino acids 45 and 46. The second domain (aa 46–150), located in the central hydrophobic core, is characterized by the Xxx-Yyy-Pro repetitive motifs. This domain is not involved in cleaved peptide production since it is included between KLK4 and matrix metalloproteinase 20 (MMP-20) cleavage site. The C-terminal domain (aa 151–180) is the hydrophilic region of the protein containing three MMP-20 catalytic sites [2,61,62].

The primary structure of the N- and C-terminal ends are highly preserved among species, while small variations were reported in the middle domain, mainly deletions or insertions of Xxx-Yyy-Pro motif [63].

The secondary structures consist of a large fraction of β-sheets, turns, and random coils with minor α-helix fractions [64,65]. The N-terminal region seems to contain beta-strand structures, and the mid-section is rich in polyproline II and β turns, while the C-terminal region displays characteristics of a random coil conformation. A Ca_2_^+^ binding site, constituted by repetitive beta-turn segment and a "beta-spiral", is also present [2,66].

To date, the AMG tertiary structure has not been well characterized, but its supramolecular assembly in nanospheres has been described [66]. AMG forms nanospheres by binding hydroxyapatite crystals with both the N-terminal and C-terminal domains. The central region forms the dense central area of the nanospheres. The function of the C-terminal hydrophilic domain is critical in the early stage of enamel formation but, during the protein secretion into the extracellular space, it is influenced by the MMP-20 catalytic cleavage.

AMG showed a quaternary structure as a function of pH (monomers at pH 3; oligomers at pH 5.5; nanospheres—self-assemblies of oligomers—at pH ≥ 6.8) and concentration [67]. It has been suggested that the N- and C-terminal domains play important roles in controlling the interactions [68], size, polydispersity [69], and hierarchical structure [70] of the nanospheres, while the central hydrophobic core promotes oligomer–oligomer binding. The N-terminal region includes a tyrosyl motif that binds acetylglucosamine and keratins, which may alter nanosphere formation and AMG’s biological role in enamel mineralization.

AMG undergoes sequential proteolysis by MMP-20 and KLK4 during enamel secretion and maturation, resulting in highly heterogeneous fragments in the enamel matrix, with a molecular weight ranging from ~5 to ~25 kDa, depending on proteolytic degradation and alternative mRNA splicing [51].

#### 3.2.2. Biology and Translational Research

Enamel matrix derivatives (EMD) have been used in periodontal disease therapy to induce the regeneration of lost periodontal tissue [71]. In dentistry, it is used for the surgical treatment of vertical bone defects that are formed around the teeth due to periodontitis. Based on the high degree of sequence homology between human and porcine enamel proteins, non-erupted developing pig premolars and molars have been employed to produce EMD. Emdogain^®^ is a commercial preparation of enamel matrix proteins composed primarily of AMG. Because of the animal origin, a batch-to-batch variability and the production of anti-EMD antibodies in the host have been reported. Two systematic reviews [3,72] have confirmed that EMD helps to restore the tissues destroyed by bacterial infection. However, the clinical trial heterogeneity imposes a certain caution in the analysis of the real treatment efficacy [72]. In addition, for a long time, the exact EMD composition was undisclosed. Now we know that the major EMD components are AMG and their fragments [73,74,75,76], with molecular weights ranging from 5 to 20 kDa [77]. The remaining portion is constituted by ameloblastin, enamelin, tuftelin, enamelysin, and enamel matrix serum proteinase 1, now officially designated KLK4 [5,78,79,80,81,82,83,84].

AMG’s biological effects are a function of the isoform/fragment employed, their concentrations, implantation site, and delivery modality. Its activity also depends, in vitro, on cell type and differentiation stage, and, in vivo, on the selected animal model [85]. For instance, in studies on vascular cells, periodontal ligament, and osteoblasts, AMG stimulates proliferation and differentiation [86], as well as transforming growth factor β (TGF-β) [87] and vascular endothelial growth factor (VEGF) release [88]. The different EMD production methods and the batch-to-batch variability might be also responsible for the different biological activity sometimes observed [44].

Regarding EMD’s regenerative potential assessed in clinical periodontology, where different tissues and cells were evaluated (guided tissue regeneration), readers may refer to the cited clinical reviews [5,72].

Focusing the discussion on bone tissue regeneration, EMD was tested on different osteoblast cell-lines such as MG63, MC3T3-E1, human osteoblast 2T9, and rat calvaria osteoblasts. Common denominators in treated cells were the increased proliferation, early differentiation, increased secretion of IL-6, type I collagen, osteoprotegerin (OPG), TGF-β1, and the inhibition of osteoclastogenesis [78,86,89,90,91,92,93,94].

Interestingly, using precursor cells such as C2C12, mesenchymal cell line, murine macrophage cell line RAW 264.7, porcine alveolar bone cells, and the stromal cell line ST2, EMD was found to stimulate osteogenic and/or chondrogenic lineages. It was reported that the osteopontin (OPN) and CBFA1/RUNX2 mRNA expression and the phosphorylation of SMAD1 may be mediated by a bone morphogenetic protein (BMP)-like peptides present in EMD [74,95,96,97,98,99,100].

Recently, Miron and coworkers demonstrated that Osteogain^®^, another commercial EMD formulation, increases ST2 pre-osteoblast attachment and osteoblast differentiation in vitro [101]. However, these results conflict with previous studies of the group of Nishihara, which have shown that EMD induces osteoclastogenesis through RANKL expression in primary osteoblasts and RAW 264.7 cells [102,103].

Results from in vivo studies in large bone defects or CSD showed that EMD is not effective in the stimulation of new bone formation [104,105,106]. On the other hand, three studies have reported a significantly higher bone fraction volume of newly formed bone trabeculae in the EMD-treated group, seven days after injury [107,108,109]. However, it seems that the best results were reported in the presence of restraints and not in large bone defects or CSD showing some osteopromotion activity in the early healing phases. It is important to emphasize that the observational periods (maximum four weeks) are very short compared to the time required for complete bone repair, which is estimated to be close to 36 months [110].

These concerns agree with those reported by Cornelini et al. on the duration of the EMD activity [111]. In fact, Emdogain^®^ is completely resorbed after four weeks and does not support either the stability of the blood clot or the space maintenance for sufficient time [112]. It is, therefore, possible to hypothesize that the short half-life and lack of dimensional stability negatively affect the release kinetics of the AMG with osteoinductive activity present in the EMD. Interestingly, Miron et al. have described that additional benefit may arise by using natural bone mineral particles pre-coated with EMD. This combination improves new bone formation after four and eight weeks post-implantation [113].

The identification of the EMD component(s) responsible for the osteoinductive activity will allow the use of synthetic peptides (standardized composition), leading to a deeper understanding of their actual effectiveness in bone tissue engineering [114]. With this aim, Iwata et al. have fractionated enamel matrix extract from porcine teeth and described an osteoinductive fraction (OFE) containing mainly proteins with a MW ranging from 20- to 23kDa [115]. This fraction has induced a mineralized nodule formation and an up-regulated osteocalcin (OCN), bone sialoprotein (BSP), and alkaline phosphatase (ALP) mRNA expression in ST2 cells without affecting their proliferation. The chromatographic separation of EMD allowed them to highlight fractions with different functions. In particular, the fractions 4–6 showed BMP-like activity, while the fractions 8–13 had TGFβ-like activity [75]. Exposure of mouse osteoblast-like cells (ST2) to fraction 3 (extracted from porcine permanent molars) reduced ALP activity, whereas fraction 2 showed an opposite effect, inducing an increased ALP activity [19].

Another research frontier was opened using recombinant AMG that showed significantly higher osteoinductive effects than EMD alone. Hoang et al. showed that recombinant porcine AMG (rP172) promotes adhesion of MG63 cells [116], and recombinant murine AMG (rM179) incorporated into a biomimetic apatite layer induced a significant increase in gene expression level of type I collagen, ALP, and OCN, as well as an enhanced cell attachment and spreading in human embryonic palatal mesenchymal pre-osteoblasts (HEPM 1486) [117]. Commercially available primary human osteoblasts exposed to rp(H)M180, a recombinant murine histidine-tagged AMG, showed about a 2-fold increase of secreted OCN with respect to the negative controls [118]. Terada et al., using an AMG-coated TNS-modified titanium surface, showed an increased ALP activity, OCN production, calcium deposition, and osteogenesis-related gene expression in RBM cells. They observed differences in the expression levels of osteoblast-specific markers between AMG-coated and uncoated TNS-modified titanium implant surfaces: an upregulation of Runx2 and BMP, which are key transcription factors mediating osteoblast differentiation, and ALP and OPN levels in RBM cells grown on the coated surface that reflected its greater capacity for inducing osteogenic differentiation. These data suggest that AMG maintains the viability of adherent stromal cells and promotes their osteoblastic differentiation [119].

An interesting aspect is the interaction between AMG and other proteins and the signaling pathways involved. AMG has been shown to exhibit several characteristics of cell adhesion proteins but, since it does not contain an RGD or other specific adhesion sequences, the involved receptor may not be an integrin [116]. Putative full-length AMG (rM180) binding partners were identified in SaOS-2 osteoblastic cells. They include chaperone molecules (HSP70 family proteins), cytoskeletal proteins (actin, vimentin, tubulin), actin-binding proteins (gelsolin, tropomyosin), proton pump proteins (ATPase), sialic acid-binding Ig-like lectins (Siglec-10), stress-inducible endoplasmic reticulum chaperone proteins (glucose-regulated protein 78, Grp78), calreticulin (CRT)), mitochondrial membrane proteins (prohibitin), and nuclear proteins (nucleophosmin and hnRNP A2/B1) [120]. Some candidate receptors are: LAMP1 in murine dental follicle cells and OCCM-30 cells [121]; LAMP1 and CD63 in human osteoblast (hFOP_1.19), murine pre-osteoblast cells (MC3T3-E1) and mouse ameloblast-like LS8 cells [122]; and Grp78 in SaOS-2 [120] and undifferentiated Human Periodontal Ligament (HPDL) cell line [123].

Previous studies have also demonstrated that recombinant full-length AMG (rM180) can rapidly move into the cell, through LAMP1 or LAMP3 (CD63)-positive vesicles, and subsequently localize to the perinuclear region in ameloblast [122], osteoblast [120,123], cementoblast, and dental follicle cells [121]. There is also evidence that enamel matrix proteins are internalized by primary human osteoblasts through clathrin-coated pits, indicating a receptor-mediated endocytosis [90].

In contrast to murine cementoblasts (OCCM-30) [85], human-derived cementoblasts treated with full-length AMG (rh174) showed an increased expression of tissue-nonspecific ALP, OCN, BSP, and mineralized nodule formation [124]. In fact, full-length AMG reduced OCCM-30 differentiation potential, featuring an increased osteopontine and a decreased osteocalcin mRNA levels. No change or a down-regulation of BSP, as well as a reduction in mineralized nodule formation, were reported [24,125,126].

### 3.3. LRAP

The Leucine-Rich Amelogenin Peptide (LRAP) is a 59-residue natural splice-variant of AMG [127]. The spliced mRNA encodes a 79-residue peptide referred to as Pro-LRAP that undergoes the telopeptide proteolytic cleavage between residue 167 and 168 by MMP-20 leading to the mature LRAP. This peptide was isolated and purified from secretory enamel matrix by Fincham and coworkers in 1981 [128], but only later was it recognized as an osteogenesis-inducing factor [17]. The 59-residue peptide is composed of the first 33 and last 26 AMG residues and is a naturally occurring product of alternative splicing of the primary mRNA transcript, with the N- and C-terminal charged regions of the full-length protein.

The secondary structure is mainly constituted by random coils in the C-terminus and N-terminus of the protein [129,130,131], even though numerical simulations suggest the presence of partially helical regions at the C-terminus (aa 48–55) and the N-terminus (aa 12–17) [131].

Yamazaki and colleagues have investigated the role of protein phosphorylation on LRAP secondary structure in the presence of hydroxyapatite and amorphous calcium phosphate. They showed that LRAP(-P) mainly consist of random coil and PPII helix or β-sheet structure, while LRAP(+P) exhibits more β-sheet and α-helix, with little random coil. With the addition of Ca^2+^, the random coil content increased in LRAP(-P), while LRAP(+P) exhibited a decrease in α-helix components. Incubation of LRAP(-P) with hydroxyapatite or amorphous calcium phosphate resulted in comparable increases in β-sheet structure. Notably, LRAP(+P) secondary structure was more affected by amorphous calcium phosphate than by hydroxyapatite, mainly showing an increase in β-sheet structure [132].

LRAP seems flexible enough to have several possible tertiary conformations.

Concerning quaternary structure, LRAP is primarily a monomer over a wide range of concentrations, pH values, salt concentrations, and in the presence of calcium. However, Ma et al. showed a hierarchical LRAP self-assembling with the formation of nanospheres, nanorods, associated nanospheres (nano strings), gel-like precipitations, and amyloid-like structures. LRAP amyloid-like supramolecular structures are assemblies containing a consistent rigid β-sheet secondary structure (residues 12–27) that seems to be essential for amyloidogenic aggregation [133]. LRAP monomers are the dominant species in solutions at pH values higher than the isoelectric point, but the solutions also contain a relatively low concentration of oligomeric species (0–16%). The differences in quaternary structures between LRAP and AMG may show which domains are important in the formation of supramolecular structures. Indeed, the missing central region is necessary for nanosphere formation since it promotes oligomer–oligomer binding.

Several studies have tried to elucidate the physiological role of LRAP in enamel formation. Its involvement in cell signaling [8,24,34,35,134], in the regulation of the calcium phosphate mineralization kinetics, and the morphology of formed crystals has been reported [25,132,135]. LRAP shares many common properties with the full-length AMG in the regulation of mineral formation in vitro: it forms nanospheres [25,136,137] and binds hydroxyapatite [138,139]. In vitro experiments using non-phosphorylated recombinant human LRAP and recombinant human AMG (rH174) showed that they have the same ability to bind calcium (i.e. 4 to 6 calcium ions per molecule), although the calcium affinity constant for the LRAP was greater than that observed for AMG [140].

#### 3.3.1. LRAP as a Cell Agonist

Some AMG isoforms do not seem to have biological activity, while others activate intracellular signaling pathways in cementum-derived cells [24,121,125] and have a great periodontal regeneration potential [5,23,141].

LRAP has been shown to be the main factor within enamel matrix derivatives to promote osteogenesis. It was reported that human and mouse ameloblast cells (LS8), after a treatment with rh58 (recombinant human LRAP) and M59 (murine LRAP), showed an upregulation of AMG, a down-regulation of the Notch1 expression, and an increased synthesis of total nitrites [140,142]. Nitric oxide (NO) is a key molecule involved in the regulation of survival, proliferation, and differentiation in many cell types. Interestingly, an increased NO level has been shown to regulate cell differentiation [143,144]; in fact, it is considered a key negative cell cycle regulator, blocking cell proliferation, a key event in the differentiation process [145]. NO inhibits collagen and proteoglycan synthesis. It also activates MMPs, mediating chondrocyte apoptosis and promoting inflammatory response in cartilage tissue. However, beneficial effects on bone metabolism by stimulating osteogenesis-related gene/protein expressions have been reported [36,146,147,148].

**Figure 3:**
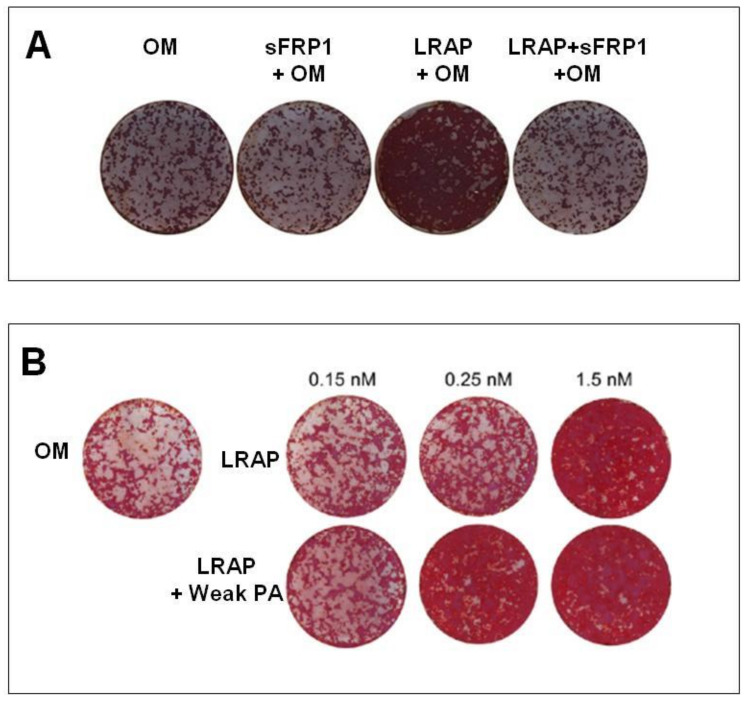
(**A**) Wnt antagonist sFRP-1 abolishes the effect of LRAP on the stimulation of osteogenesis of ST2 cells. Alizarin red staining for analysis of mineral deposition two weeks after osteo-induction (Reproduced with permission from [35]); (**B**) Differentiation of ST2 bone marrow stromal cells treated with the LRAP signaling molecule. Two weeks after bone induction, mineral deposition was assayed with alizarin red staining. (Reproduced from [36]. This is an unofficial adaptation of an article that appeared in an ACS publication. ACS has not endorsed the content of this adaptation or the context of its use.)

In Table 2, we list the papers that have investigated the ability of LRAP to induce synthesis of bone mineralized extracellular matrix [8,34,35,36]. Warotayanon and colleagues were the first to test and hypothesize that LRAP could be an alternative pharmacological agent in craniofacial bone tissue and skeletal defects because it induces the expression of bone marker genes in osteogenic-induced ES cells [8,34]. On murine bone marrow-derived stromal cells (ST2), LRAP, at a concentration of 10 ng/mL, increased Runt-related transcription factor 2 (Runx2) and osteocalcin without affecting cell proliferation. At this concentration, LRAP in combination with osteogenic medium allowed a significantly higher mineral deposition two weeks after treatment (Figure 3A) [35]. In a recent work of Newcomb and colleagues, ST2 cells osteogenesis was stimulated with LRAP seeded into an amphiphilic peptide-based scaffold. They have reported that LRAP has a dose-dependent activity, showing both an increased calcium phosphate deposition (Figure 3B) and an increased expression of osteogenic markers, such as *Runx2, Osx* (osterix), *Dlx5* (distal-less homeobox 5), and *Col2a1* (collagen type 2 alpha 1), comparable with the control group (C2C12-immortalized mouse myoblast cell line) treated with BMP-2, further underlying the role of LRAP in osteogenesis [36].

The study of Matsuda et al. investigated the effect of chemically synthesized LRAP (csLRAP) on chondrogenic or osteogenic differentiation of the ATDC5 and the MC3T3-E1 cells. Both chondrogenic and osteoblast cells showed a significant suppression in cell number in the presence of csLRAP at 10 μg/mL compared to the control. Furthermore, the intensity of alcian blue staining in chondrogenic cells and alizarin red staining in osteoblast cells were significantly increased in the presence of csLRAP at a dose of 10 μg/mL after four weeks of culture. Chondrogenic or osteogenic differentiation marker genes, including *Sox9*, *Col2a1*, *Col10a1*, *Runx2*, *Alpl* (alkaline phosphatase), and *Col1a1*, were upregulated in the presence of csLRAP after one week of culture. Interestingly, all these effects were suppressed in the presence of LAMP-1 antibody. Together, these results suggest that csLRAP could promote osteogenic and chondrogenic differentiation in vitro and that LAMP-1 may be involved in the differentiation and proliferation of these cells [37]. In fact, there is a previous report that describes LRAP as a negative regulator of osteoclastogenesis [26], underlining the role of LRAP in bone regeneration.

Of note, LRAP and the full-length AMG showed an opposite effect also on cell proliferation, although in two different cell models. Wen X et al. reported that LRAP has no effect on the proliferation of ST2 cells [35]; on the contrary, human recombinant full-length AMG has been shown to increase the proliferation rate of human bone marrow mesenchymal stem cells [149].

There are no in vivo studies that demonstrate the effectiveness of recombinant LRAP alone in bone regeneration. However, EMD fraction rich in LRAP and ameloblastin, that the authors call “pool 7”, was tested and injected in the sub-periosteal calvaria of mice [84]. Authors reported an increased induction of phospho-SMAD, Osterix, and VEGF-α. In this in vivo study, the solution also containing LRAP was administered daily for five days, and the stimulus was then regenerated at each administration. Of particular interest is the induction of the transcription factor Osterix, a key regulator in bone formation [150]. LRAP increases the transcriptional level of key genes such as COLL1A1 (collagen, type I a1), and recently it has been demonstrated to directly regulate the angiogenetic factor VEGF-α, thus linking bone formation to angiogenesis [151]. Regarding the other higher molecular weight components of EMD, Villa O. et al. indicate that they might be related to an angiogenic effect through the stimulation of VEGF release and might modulate wound healing through the expression of IL-6 [152]. These findings indicate that some components of “pool 7” of commercially available EMD can activate BMP signaling within the periosteum, in the newly induced osteoblasts, as previously suggested by Zhao M et al. [153]. Obviously, these results should be considered in relation to the presence of osteoblastin. In fact, other authors describe this protein as a positive modulator of BMP signaling [153].

#### 3.3.2. LRAP Candidate Receptors

For a long time, it was unclear how AMG interacts with target cells to increase the expression of the osteogenetic markers that we have previously listed. Although specific receptors for each component of the enamel matrix (including LRAP) remain to be consolidated, some evidence has suggested that cellular uptake of enamel matrix proteins by different cell-types involves receptor-mediated endocytosis.

Before 2016, there are no studies evaluating the interaction between LRAP and cell membrane proteins. All researchers investigated the EMD fractions or the recombinant full-length AMG.

In a pioneering work of Wang HJ et al., different proteins, such as the eukaryotic translation elongation factor 2 (Eef2), the fasciculation and elongation protein zeta 1 (Fez1), and the small nuclear RNA-specific Sm-like protein splicing factor (Lsm10), were described as possible LRAP binding proteins [27]. It is well established that full-length AMG can bind membrane proteins such as LAMP1 and LAMP-3 (CD63), two proteins associated with endosomal/lysosomal membrane and Annexin A2 (ANXA2), not only on ameloblasts [154] but on different cell lines [90,121,122,142]. If cell surface LAMP-1 was blocked with an antibody, the sorting of exotic vesicles changes increasing the level of intracellular recombinant AMG [124]. An autocrine interaction between LAMP-1 and rM150 (recombinant mouse AMG) was also observed in osteoblastic cells lines [120,121,122]. In fact, in this case, authors demonstrate that rM150 induced an up-regulation of the LAMP1 receptor. The key relationship between LRAP and LAMP-1 is demonstrated by the fact that several differentiation marker genes in MC3T3-E1 cells were increased by synthetic LRAP and, conversely, were suppressed in the presence of LAMP-1 antibody [142]. The involvement of LAMP-1 as LRAP receptor was further pointed out by Matsuda and coworkers [37].

In addition, LAMP-3 was shown to bind M180 proteins [122]. As reported by Amin et al., some component(s) of the commercial preparation of EMD (Fraction A and Fraction C) bind(s) only PDL cells that express the receptors(s) that mediates direct interaction. These proteins were subsequently internalized and transported to the perinuclear region of the cells with a diffuse cytosolic co-localization with LAMP-1 positive lysosome-like structures [73]. By using immortalized murine cementoblasts (OCCM-30), Martins et al. have demonstrated that LRAP binds LAMP-1, LAMP-3 and, for the first time, Flotillin-1 (Flot-1) [85]. Flot-1 is a ubiquitously expressed and highly conserved protein, which has been suggested to be involved in a variety of cellular processes, such as signal transduction, endocytosis, phagocytosis, cellular trafficking pathways, cell adhesion, and regulation of actin cytoskeleton. Flot-1 is constitutively associated with lipid rafts, small membrane microdomains enriched in cholesterol and glycosphingolipids [155,156]. Lipid rafts function as platforms for various cellular processes, including signal transduction and membrane trafficking [157,158]. An involvement of the ERK/MAPK pathway in cementoblast cells was suggested as an LRAP signaling pathway in 2004 [24]. In particular, it was shown that LRAP-Flot-1 interaction was also involved in signal transduction processes and endocytosis originated from lipid rafts [85].

It is now well accepted that the activation of the canonical Wnt signaling pathway [159] by LRAP induces the osteogenic differentiation of mouse embryonic stem cells (mES), and this activation is more powerful than exogenous Wnt3a in terms of Osx and BSP expression levels and mineral deposition [8,35]. Recently, Newcomb et al. have confirmed that the Wnt canonical pathway is activated by LRAP at a concentration of 10 ng/ml. They showed that a higher concentration does not exert any increase in the expression of osteogenetic markers because the Wnt signaling cascade is saturated [36].

### 3.4. TRAP

Tyrosine-Rich Amelogenin Peptide (TRAP) is a 5.1 kDa isoform of EMD generated from the full-length AMG through proteolytic cleavage. The amino acid sequence is the following: MPLPPHPGHPGYINFSYEVLTPLKWYQNMIRHPYTSYGYEPMGGW.

The biological role of TRAP in periodontal cells is still matter of debate. In fact, it does not seem to have any effect, not even a suppressive effect, on alveolar bone or the periodontal ligament [28,84]. On the other hand, it was reported that chemically synthesized TRAP stimulates angiogenic differentiation of human periodontal ligament stem cells [29,30,160] and angiogenesis in human gingival fibroblasts [161].

The angiogenic property [162,163] and expression of angiogenesis-related proteins in endothelial cells (ECs) [88,164,165,166] have been widely documented in several in vitro and in vivo studies. However, EMD-derived fraction containing TRAP seems more active than TRAP in stimulating angiogenesis [28]. A plausible explanation of this biological effect was given by Jonke et al. in 2016 [161]. TRAP, isolated from EMD or chemically synthesized, upregulates the expression of adhesion molecules as ICAM-1 and E-selectin, localized on the endothelial cell surface [167,168]; VEGF, that plays a central role in angiogenesis [169]; kinase insert domain receptor (KDR), localized on the endothelial cell surface, with an important role in endothelial cell differentiation; and the FMS-like tyrosine kinase receptors 1 (FLT-1) [170].

Von Willebrand factor (a multimeric glycoprotein known for its contribution to the hemostatic process) upregulation by TRAP was reported for the first time by Amin and colleagues in 2014, and in 2016 by Jonke at al. [29,161]. These authors suggest that TRAP might improve interaction between different cell types and promote VEGF release by resident fibroblasts and VEGF response by endothelial cells during the process of wound healing. Interestingly, TRAP seems to be able also to increase the expression of VEGFR2, an early endothelial marker gene (a tyrosine kinase receptor for the VEGF ligand) on endothelial cells, together with the late genes Tie-1 and Tie-2, two tyrosine kinase receptors for angiopoietin that are exclusively expressed by endothelial cells [171], and VE-cadherin, an endothelial cell adhesion molecule in human periodontal cells. The angiogenic effect was also demonstrated by using the chicken embryo chorioallantoic membrane assay [29]. The proliferation/viability of endothelial cells was significantly decreased after treatment with TRAP, peptides with either 43 or 45 aminoacidic residues separated and purified from EMD, and synthetic TRAP at a concentration of 100 μg/ml [161].

The low-molecular-weight EMD fraction, containing TRAP, has an osteoblastic effect on PDL fibroblasts [160]. The authors have found increased *RUNX2*, OPN, OCN, BSP gene expression levels and an increased alkaline phosphatase activity.

**Figure 4:**
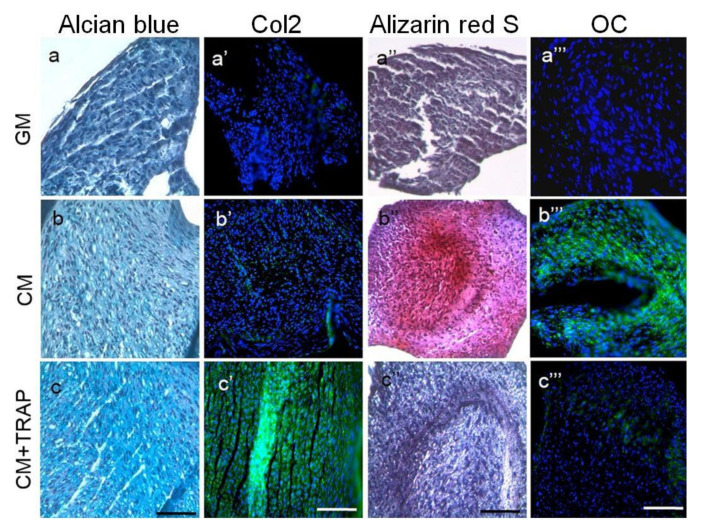
Histology indicates that 10 μg/ml TRAP promotes chondrogenic differentiation and suppresses hypertrophic mineralization. Labels on individual panels refer to culture media type: (**a**–**a**’’’) GM; (**b**–**b**’’’) CM; (**c**–**c**’’’) CM+TRAP. Note the intense bright blue staining of TRAP-treated cell pellets stained with alcian blue (**c**, indicative of glycosaminoglycans present in the ECM) and the corresponding lack of Alizarin red staining (**c**’’, indicative of minimal calcium deposition). Note also the green fluorescent staining of TRAP-treated cell pellets immuno-stained with Col2 (**c**’) and the corresponding lack of OCN staining (**c**’’’). Alcian blue and alizarin red sections were counter-stained with Harris Haematoxylin (purple nuclei); Col2 and OCN sections were counter-stained with Hoechst dye (blue nuclei). Scale bar 100 μm. (Reproduced from [38], an open access article distributed under the terms of the Creative Commons CC BY license.)

In adult primary human articular cartilage cells (HACs), TRAP seems to suppress hypertrophic mineralization and concomitantly promotes chondrogenic differentiation through both early and late chondrogenic gene induction (i.e., SOX9, *COL2A1*, and ACAN). These results were observed when cells were cultured in chondrogenic conditions supplemented with TRAP (10 μg/mL) but not in control medium (Figure 4) [38]. These results are in agreement with previous reports in which they demonstrated that TRAP suppresses bone-forming activity through Smad6-mediated *RUNX2* inhibition [28]. In the same year, Tanimoto and colleagues showed no significant effects on mineralization and expression of osteogenic markers of TRAP on human periodontal ligament cells (HPDLs) [124].

### 3.5. SP (Synthetic Peptide)

The bioactivity of EMD varies from batch to batch and has been reported to be antigenic and to induce the production of anti-EMD antibodies in the host. To overcome these issues, Kim et al. analyzed the active sites for eosinophilic round bodies (ERBs) binding in EMD-associated proteins using matrix-assisted laser desorption ionization time-of-flight mass spectrometry. They identified a 7 amino acid sequence (WYQNMIR) in AMG that corresponds to a portion of the AMG exon 5. This sequence, named SP, has a molecular mass of 1118 Da, and it is less likely than EMD to elicit an immunological response [31].

The use of a synthetic peptide would allow one to avoid the use animal derivatives and would be suitable in terms of safety and reproducibility. Scientific evidence on its potential use in tissue engineering and regenerative medicine appeared in the literature only in 2009 (Table 2).

Periodontal ligament fibroblast treated with SP had an increased expression of genes related to osteogenesis, such as BMP receptor type 1 A, BMP4, osteonectin, BMP receptor type1 B, osteocalcin, as well as ALP activity, and intracellular calcium deposition with a parallel decreased expression of fibroblast growth factor receptor-like protein 1 [39,42].

**Figure 5:**
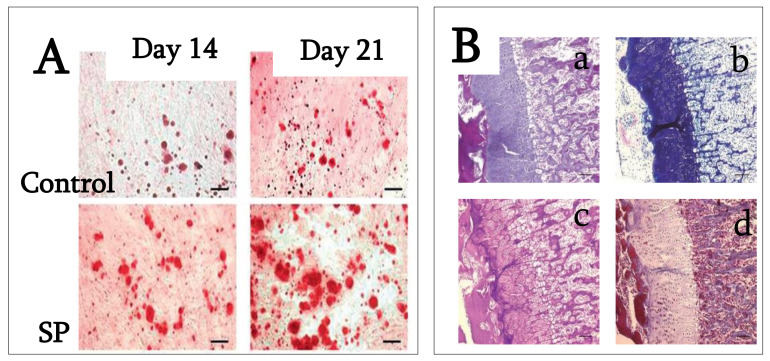
(**A**) PDLSC cultures were stained with alizarin red S after 14 and 21 days of cultivation in osteogenic medium with and without 100 ng/mL SP. Calcium deposition on days 14 and 21 was higher in the presence of SP than in its absence. Bar = 100 mm. (Reproduced with permission from [43]). (**B**) Endochondral ossification and bone formation are observed in the backs of rats 14 days after injection in rats injected with 15 mg/mL concentration of synthetic peptide (**a**): hematoxylin and eosin stain). Metachromasia is demonstrated at cartilage tissue with toluidine blue (**b**). Both cartilage and bone tissue are positive for PAS (**c**), but negative for Masson trichrome (**d**). (**a**–**d**, original mag. ×5; bar: 0.1 mm; reproduced from [40].)

These data indicate that SP is effective in periodontal tissue regeneration, suggesting that it may convert human periodontal ligament fibroblasts to bone-forming cells and act as a growth factor. On rat bone marrow cell cultures, SP increases cell proliferation, adhesion, and chemotaxis, reporting significantly higher alkaline phosphatase activity and Ca^2+^ deposition after 7 and 14 days of treatment [41]. Kato and colleagues investigated SP’s effect on human periodontal ligament stem cells (PDLSCs), concluding that it can enhance osteoblastic differentiation at early stages of differentiation and can be effective in the initial stage of periodontal tissue regeneration. The authors showed that cell proliferation is significantly increased in the presence of SP in both normal and osteogenic medium. In SP-supplemented osteogenic medium, the expression of osteonectin and osteocalcin mRNA, ALP activity, the number and size of calcified nodules, mineralization, and osteocalcin production were significantly higher than non-supplemented osteogenic medium (Figure 5A) [43].

Thus, SP apparently promotes the mineralization of extracellular matrix on PDLSCs, suggesting that SP promotes PDLSC proliferation, the expression of mineralization markers, and the formation of calcified nodules highlighting the potential of SP in periodontal tissue regeneration. In 2014, Katayama et al. synthesized the same oligopeptide derived from EMD to evaluate its contribution to periodontal tissue regeneration. They investigated the SP effects on cell proliferation and osteoblastic differentiation of human mesenchymal stem cells (MSCs). SP (0 to 1000 ng/mL) promoted cell proliferation, osteoblast differentiation, and matrix mineralization, probably through the ERK signaling pathway [44].

These results indicate that SP would be useful for periodontal and bone tissue regeneration because the promotion of MSCs proliferation and differentiation is described as being essential in these processes. Moreover, SP is unlikely to be antigenic and it can be produced synthetically. However, the molecular mechanisms by which SP acts on cells are not yet clearly understood and must be further investigated. Only one paper has investigated SP’s ability to induce the extracellular matrix mineralization and bone tissue formation in animal models. SP seems to produce heterotopic ossification in rats when injected under the skin, with endochondral ossification and bone formation within 14 days from the injection (Figure 5B) [40].

### 3.6. C11 (Amelogenin C Peptide, AMG-CP)

A variety of studies suggest that the C-terminal tail of AMG (C11) plays an important role in enamel biomineralization [172]. Zhu et al., using AMG mutated variants (P156T and P164T), demonstrated that the substitution of proline with threonine at position 156 or 164 displayed a significantly lower affinity to HAP, suggesting that these 2 C-terminal prolines are important for optimal adsorption of AMG protein to HAP. The prolines appear to be essential conformation determinants that alter the accessibility of AMG C-terminus to apatite, which is related to the growth of apatite crystals and enamel development [173].

It is noteworthy that prolines are found adjacent and upstream to all identified MMP-20 cleavage sites in the AMG sequence [174]. Proline is the only cyclic amino acid with a pyrrolidine ring that restricts the conformation range of adjacent residues. The regularly spaced prolines are presumably important in maintaining the extended chain conformation of proteins. The proline residues in the AMG C-terminus are highly conserved across many species, suggesting a functional role for the initial processing of AMG and AMG–mineral interactions.

C11 stimulates the proliferation of human cementoblast-like cell line [175], hAD-MSCs, and hBM-MSCs [32,33], enhancing the phosphorylated ERK1/2 signaling in both cell lines. These effects are inhibited by an anti-LAMP-1 antibody, a condition that further demonstrates the importance of proteins associated with the endosomal/lysosomal membrane in the signal regulation of these peptides [33,175].

**Figure 6:**
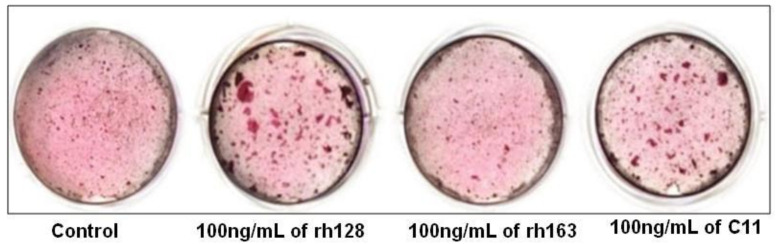
Effect of amelogenin fragment treatment on the mineralization activity of HCEM cells during osteogenic differentiation. Intensity of alizarin red staining in HCEM cells increased following treatment with rh128 or C11 peptide, but there was no obvious difference following treatment with rh163. (Reproduced with permission from [46].)

Regarding C11’s biological role in osteogenic differentiation, the available data indicate that human cementoblast osteogenic differentiation was significantly enhanced by rh128, AMG fragments lacking the N-terminus, and C11, while rh163, AMG fragments lacking the C-terminus, had no significant effect. This indicates the possible utility of C11 in periodontal tissue regeneration (Figure 6) [46]. On the contrary, Awada et al., by using similar C11 concentrations, have found an increased proliferation of mouse MC3T3-E1 cells, but no substantial effects on osteogenic differentiation [45].

Overall, these studies suggest the need of future investigations to elucidate C11 molecular mechanisms, by considering also possible differences due to different experimental protocols.

## 4. Conclusions and Future Perspectives

Since AMG’s discovery, over 1500 articles have been published, and proteins and peptides extracted from enamel matrix have been widely used in the last 25 years in regenerative surgery as the support tissue of dental elements, and a total of 132 clinical trials have been published. However, today the effectiveness of these products in promoting bone formation is not clear, and is even discordant in some cases.

In this study, we investigated AMG fragments that have aroused considerable interest in stimulating biomineralization. No clinical studies have verified the use of these peptides in guided bone regeneration procedures. On the basis of in vitro evidence reported here, SP appears to have a significantly higher osteoinductive capacity than LRAP, TRAP, and C11, with a greater tendency to induce osteoblast differentiation and proliferation of non-specialized connective cells and mesenchymal stem cells.

Although LRAP shares many of the biochemical domains with full-length AMG protein, it seems to be more effective in stimulating the formation of mineral nodules through the expression of *Runx2*, *Osx*, *Dlx5*, type I collagen, and the production of VEGF1 and IL6. Interestingly, it was also described as a negative regulator of the osteoclastogenesis and adipogenesis. Furthermore, LRAP can play a key role in promoting osteogenesis through the regulation of endosomal-osteoblastic activity and activation of the Wnt canonical signaling pathway. In contrast, TRAP does not appear to have significant effects on bone production but its binding to ICAM-1, E-selectin, KDR, and FMS-like tyrosine kinase receptors 1 stimulates angiogenetic activity and the production of VEGF by fibroblasts.

In different studies, LRAP and TRAP were isolated from animal tissue. This approach introduced a variability in the degrees of purity and titration of peptides used.

AMG C-terminal fragment showed a clear ability to induce proliferation in cells found in periodontal ligament and alveolar bone (i.e., mesenchymal stem cells and osteoblasts), but there are divergent results on its ability to induce bone differentiation. This suggests the need for future investigations on the molecular mechanisms behind the effects of the C-terminal region of the AMG peptide. Yet, there is a potential application of the C-terminal region of the AMG peptide in mesenchymal stem cells, osteoclasts, and other cell-types present in bone formation, which needs to be analyzed more deeply in vitro and in vivo. In fact, several authors have confirmed that the response stimulated by AMG peptides depends strongly on the type, species of origin, and cells treated.

The collected results on SP show that it is as an effective tool in periodontal and bone tissue regeneration. Of note, given the heterogeneity of cell types, culture systems, and molecules used, the information collected in this review should be considered with caution. However, being synthetic peptides, the higher degree of purity and the composition standardization represent an important value-added with respect to AMG from animal sources.

Overall, the use of AMG-derived peptides has influenced researchers, leading to over 50 relevant publications in the last 3 years coming almost exclusively from the dentistry area and related sciences.

We believe that the interest in AMG-derived peptides will grow soon and their potential application in bone regeneration will be further investigated. However, their possibly bright future in bone regeneration will strongly depend on the elucidation of their featured conformations and the understanding of receptor interactions and signal transductions. The use of synthetic or recombinant peptides, with a known composition, will surely increase experiment reproducibility and result consistency. However, the most interesting and reliable knowledge on AMG-derived peptides’ aptitude to induce the production of mineralized extracellular matrix will come from in vivo preclinical experiments, which are lacking.

## Figures and Tables

**Table 1:** Articles excluded from the systematic review. The table reports the main reason for the exclusion.

First Author	Peptide	Main Exclusion Criteria	Ref.
Boabaid et al. (2004)	LRAP	Osteoblast differentiation not investigated	[24]
Le Norcy et al. (2011)	LRAP	Bone formation not investigated	[25]
Hatakeyama et al. (2006)	LRAP	Bone formation not investigated	[26]
Wang et al. (2006)	LRAP	Bone formation not investigated	[27]
Amin et al. (2012)	TRAP and LRAP	No recombinant peptides	[28]
Amin et al. (2014)	TRAP	No recombinant peptides	[29]
Amin et al. (2011)	Enamel matrix derivative peptides	Bone formation not investigated	[30]
Kim et al. (2005)	Enamel matrix derivative peptides	No recombinant peptides	[31]
Ando et al. (2018)	C11	No recombinant peptides	[32]
Kunimatsu et al. (2018)	C11	Osteoblast differentiation not investigated	[33]

**Table 2:** Articles dealing with the effect of LRAP, TRAP, SP, and C11 on bone regeneration included in the systematic review.

First Author (Year)	Peptide	Cell/Cell Line	Concentration	Time Point	Main Results	Ref.
Warotayanont et al. (2008)	LRAP	RW4 and AMEL^-/-^ ESCs	10 ng/mL	10 and 20 d	The addition of exogenous LRAP significantly increases the mineral deposition and the expression of BSP and Osx.	[34]
Warotayanont et al. (2009)	LRAP	RW4 and MC3T-E1	10 ng/mL	4, 6 hand 20 d	LRAP increases the level of Wnt agonist(s) and induced an up-regulation of Osx and BSP of EB cells. The Wnt antagonist sFRP-1 blocks LRAP-mediated osteogenesis.	[8]
Wen et al. (2011)	LRAP	ST2 and MC3T3 cells	10 ng/mL	14 d	LRAP treatment elevates the Wnt10b expression level and promotes osteogenesis of mesenchymal stem cells.	[35]
Newcomb et al. (2016)	LRAP	ST2	0.15 nM, 0.25 nM and 1.5 nM	14 d	Gene expression was similar between LRAP and BMP-2 treatment. LRAP enhanced osteo-differentiation through the activation of the canonical Wnt/β-catenin signaling pathway.	[36]
Matsuda et al. (2017)	LRAP	MC3T3-E1 and ATDC5	10 ng/mL	7, 14, 28 d	LRAP could promote “in vitro” osteo-chondrogenic differentiation. LAMP-1 may be involved in the differentiation and proliferation of these cells.	[37]
Amin et al. (2016)	TRAP	HACs	1, 10, 50 and 100 μg/mL	21 d	TRAP suppresses hypertrophic mineralization and concomitantly promotes chondrogenic differentiation of HACs.	[38]
Kawanaka et al. (2009)	SP	HPdLF	1, 10 and 100 ng/mL	7 d	The mRNA content of BMPR1A was increased in HPdL F cultured with synthetic peptide. SP might convert HPdLF to bone-forming cells.	[39]
Hida et al. (2010)	SP	In Vivo study (rats)	0.3, 3, 7.5,15 and 30 mg/mL	1, 3, 5, 7, 14 d	The synthetic peptide combined with an extended-release scaffold seems to produce hard tissues, such as cartilage and bone.	[40]
Yasui et al. (2012)	SP	RBMCs	20, 100, 500 and 1000 ng/mL	7, 14 d	SP facilitates cell proliferation and induces differentation into osteoblast.	[41]
Taguchi et al. (2012)	SP	HPdLF	5, 20, 100, 200 or 500 ng/mL.	28 d	SP accelerated calcification, increases ALP activity and OCN production.	[42]
Kato et al. (2013)	SP	PDLSC	100 ng/mL	2, 3, 5, 7, 21 d	SP enhances the formation of calcified nodules and osteocalcin production.	[43]
Katayama et al. (2014)	SP	MSCs	0. 1, 10, 100 and1000 ng/mL	7 and 14 d	SP promotes cell proliferation, osteoblast differentiation, and mineralization in human MSCs.	[44]
Awada et al. (2017)	C11	MC3T3-E1	0, 100, or1000ng/mL	7, 14, 21 d	Enhanced cell proliferation, but no difference with control group in terms of osteogenic differentiation and expression of ALP and BSP was observed.	[45]
Kuminatsu et al. (2017)	C11	HCEM	0, 10,100 or 1000 ng/mL	1, 7, 14, 21 d	Osteogenic differentiation was significantly enhanced by treatment with rh128 and C11 peptide but not with rh163.	[46]
